# *CORR* Insights^®^: Coronal Limb Alignment and Indications for High Tibial Osteotomy in Patients Undergoing Revision ACL Reconstruction

**DOI:** 10.1007/s11999-013-3246-6

**Published:** 2013-08-28

**Authors:** John P. Albright

**Affiliations:** Department of Orthopaedic Surgery and Rehabilitation, The University of Iowa, 200 Hawkins Dr., Iowa City, IA 52242 USA

## Where Are We Now?

The current study by Won and colleagues concluded that patients undergoing revision ACL surgery have a greater incidence of significant varus alignment compared with patients undergoing primary ACL reconstruction. The authors also observed that this varus alignment was associated with meniscal pathology and degenerative changes of the knee. The report of varus alignment provides an interesting observation that goes a step beyond the findings of the Multi-center Anterior (C)ruciate Revision Study (MARS) [1], but leads to more questions than answers. The article does not tell us about the results of performing a high tibial osteotomy at the time of the revision ACL. It merely points out that these patients are potential candidates for such a procedure.

The strength of the manuscript is that it reminds us that varus malalignment is a potential variable to be mindful of. It also serves to warn surgeons they should be aware of possible failure of the graft, and continuing pain related to medial compartment degeneration. Won and colleagues’ attention to detail in establishing an effective protocol for the long-leg films is important because standing long-leg radiographs are fickle in terms of being able to demonstrate consistently the true amount of coronal plane alignment.

## Where Do We Need to Go?

Kim and colleagues [3] reported that aside from extreme cases with varus thrust (without medial compartment arthritis) the stability and functional scores were not adversely affected by primary varus alignment. Kim and colleagues [3] also reported that aside from extreme cases where varus thrust is noted, (in addition to the post meniscectomy changes) the radiographic features of unicompartmental osteoarthritis are insufficient to be associated with graft failure.

Another MARS study [2] indicated that a significant number of revision surgeries were related to technical difficulties in graft tunnel placement in the primary procedure. This raises a number of questions. Was the degenerative process and progressive varus at fault in causing the ligament failure in the other revision surgeries not related to graft tunnel placement? Which patients need proximal tibial osteotomies at the time of revision ACL surgery? Should a high tibial osteotomy be performed to avoid postsurgical medial joint line pain? Will concomitant realignment increase the chance of establishing stability or would staging the two procedures not only eliminate the risks associated with the combined procedure in a significant number of patients, but also prolong the function of these patients by putting off a realignment procedure with a time-and-demand-limited survival curve?

## How Do We Get There?

We need to perform a large prospective case-control study observing primary ACLs over a long period of time, and separately analyzing those who have initial varus with intraarticular pathology from patients who do not. We need to conduct followup analysis to determine whether the varus indeed increases the risk of ligament failure. Such a study should also observe the progression of osteoarthritis and secondary development of varus deformity so we could get a better picture of the natural history.

Another study that would help fill in our current knowledge gap would be examining the existence of medial compartment osteoarthritis in an ACL deficient knee that has already had a failure of a primary ACL surgery. It should be determined, in the setting of a large randomized controlled trial, whether those patients who undergo a high tibial osteotomy at the same time as ACL revision surgery will fare better than those who have an ACL revision without an osteotomy.

This should be a very well-defined study group with radiograph-based alignment protocol similar to the one used in the current study. How much varus is necessary to change the odds ratio for ACL revision alone? Is varus thrust indeed the only game changer? Will staging the stabilization and the realignment procedure provide the patient with a greater number of years of satisfactory function? These important questions remain unanswered.
